# Understanding the Gendered Impact of COVID-19 on Young Self-Employed Nigerian Women and Coproducing Interventions That Foster Better Systems and Well-Being: Protocol for a Multimethods Study

**DOI:** 10.2196/69577

**Published:** 2025-05-30

**Authors:** Iyeyinka Kusi-Mensah, Aarati Taksal, Joshua Akinyemi, Oluwatomisin Owoade, Funmilola M OlaOlorun, Ade F Adeniyi, Olayinka Egbokhare, Olusade Taiwo, Oluwabukola Adeoye, Rita Tamambang, Adeola Afolayan, Chuka Ononye, Olafunmilayo Adebukola Akinpelu, Srividya N Iyer, Olayinka Omigbodun

**Affiliations:** 1 Centre for Child and Adolescent Mental Health College of Medicine, University of Ibadan Ibadan Nigeria; 2 Douglas Hospital Research Centre Montreal, QC Canada; 3 Department of Epidemiology and Medical Statistics College of Medicine, University of Ibadan Ibadan Nigeria; 4 Abeni Prints, Textile Design Company Ibadan Nigeria; 5 Department of Community Medicine College of Medicine, University of Ibadan Ibadan Nigeria; 6 Department of Physiotherapy College of Medicine, University of Ibadan Ibadan Nigeria; 7 Biomedical Communication Centre College of Medicine, University of Ibadan Ibadan Nigeria; 8 Development Agenda for Western Nigeria (DAWN) Commission Ibadan Nigeria; 9 Oyo State Ministry of Women Affairs and Social Inclusion Ibadan Nigeria; 10 Department of Psychiatry McGill University Montreal, QC Canada; 11 Department of Psychiatry College of Medicine, University of Ibadan Ibadan Nigeria

**Keywords:** COVID-19 pandemic, low- and middle-income countries, self-employed women, resilience, well-being, coproduction, Nigeria

## Abstract

**Background:**

The COVID-19 pandemic has had disproportionate economic and health impacts on self-employed workers in Nigeria, particularly self-employed women and youth. Though uniquely different, the COVID-19 pandemic shares similarities with events such as childbirth, family, and health emergencies. Self-employed young women lack adequate support structures to cope with disruptive life events, which have negative consequences for their well-being. This is concerning, as 86% of women in the Nigerian labor force are self-employed.

**Objective:**

The project’s first objective is to conduct a gendered situational analysis to address the question of how the COVID-19 pandemic and other life events affect the paid and unpaid work and the physical, mental, and social well-being of self-employed young women in Nigeria; their strategies for coping with such events; and how their experiences compare with those of self-employed young men. Informed by this analysis, the second objective is to coproduce and pilot-test a gender-transformative intervention that integrates social protection and promotes well-being.

**Methods:**

This multimethod project has 3 components. The first is a situational analysis of the impact of the pandemic and other significant life events on the work and well-being of self-employed young women vis-à-vis self-employed young men. This involves qualitative interviews with approximately 60 self-employed young women and men and a digital storytelling initiative to represent some of these stories in video format. Secondary data analysis of the Nigerian General Household Survey and the COVID-19 Longitudinal Phone Survey will be conducted. Furthermore, a scoping review of the impact of significant life events, including the COVID-19 pandemic, on self-employed workers in low- and middle-income countries will be conducted. The second component is the coproduction of interventions involving qualitative interviews with self-employed young women, members of their support network, and policy makers to find out their views on how to support self-employed women. It also entails an analysis of policies relevant to self-employed women in Nigeria and theory of change workshops to create a map for achieving the long-term goal of improving their resilience. Furthermore, a systematic review of interventions to improve the job quality and well-being of self-employed workers will be conducted. The third component is a pilot of the coproduced interventions in a quasi-experimental study involving 300 participants to assess feasibility, acceptability, cost, and potential effectiveness.

**Results:**

This project was funded in October 2022. Data collection for the project commenced in May 2023 and will end in November 2025. Data collection for the situational analysis and coproduction of intervention phases have been completed while the pilot of intervention packages is underway.

**Conclusions:**

This project will advance knowledge of the impact of the COVID-19 pandemic and other significant disruptive life events on the work and well-being of self-employed young Nigerian women and provide coproduced solutions to mitigate their effects.

**International Registered Report Identifier (IRRID):**

DERR1-10.2196/69577

## Introduction

### Background

The COVID-19 pandemic has had disproportionate economic and health impacts on self-employed workers. In high-income countries, self-employed workers lost more earnings and jobs than employees [1,2]. In low- and middle-income countries (LMIC), the economic consequences of the pandemic were even greater. With little to no social protection, paid sick leave, savings, or access to credit, many self-employed workers in LMIC continued to go out to work at the height of the pandemic, exposing them to an increased risk of infection [3]. They also faced adverse mental health consequences, including fear of infection and income-related anxieties [4,5].

According to the International Labour Organization, self-employed persons are workers whose remuneration directly depends on the profit from the goods and services they produce. They include employers, own-account workers, members of producers’ cooperatives, and contributing family workers [6]. Self-employment accounts for 70% of employment globally [7]. LMIC generally have a higher proportion of self-employed workers in their labor force [8]. In Nigeria, 80% of workers and 86% of women in the labor force are self-employed, making it an apt setting to examine the impacts of the COVID-19 pandemic on self-employed workers [9,10]. Self-employed workers in Nigeria usually work in agriculture, wholesale and retail trade, catering and food services, fashion design, machinery repair, the digital economy, etc. Many self-employed workers operate in the informal economy, where economic activities are unregulated by the government and other formal institutions of society [11].

Self-employed women face certain disadvantages in the labor market. They have fewer employees and lower earnings and profits [12-15]. A report from the Organisation for Economic Co-operation and Development [16] suggested that compared to self-employed men, self-employed women earn less and have smaller businesses because they have less access to capital, enter into less competitive sectors, and are more likely to operate in the informal economy. The COVID-19 pandemic heightened these disadvantages. United Nations Women [17] reported that 25% of self-employed women lost their jobs during the pandemic compared to 21% of self-employed men. Furthermore, the US Global Leadership Coalition revealed that in every region of the world, female-owned businesses experienced higher closure rates during the first year of the pandemic compared to male-owned businesses [18]. In Nigeria, about 44% of women-led businesses closed during the pandemic compared to 33% of men-led businesses [19].

Data from Ibadan in the southwestern part of Nigeria collected in 2021 revealed the pandemic’s heightened impacts on self-employed women [20]. The data came from 29 semistructured interviews and 504 questionnaires administered to self-employed young men and women from diverse sociodemographic backgrounds working in various sectors. Many self-employed workers experienced financial hardship, resulting in distress. The lockdown prevented 38.8% of self-employed young women from doing business-related tasks and forced 23.6% of women (vs 17.8% of men) to run their businesses from home. Fewer women than men (32.5% vs 40.6%) continued going out to work during lockdowns. During the pandemic, 65.8% of women reported facing “much” or “a lot” of financial hardships. Among self-employed youth who had started their businesses before the COVID-19 pandemic, 3.5% of 255 women (vs 7.6% of 197 men) were able to start a new revenue stream to cope with the effects of the pandemic, and only 1 woman received government monetary assistance.

Though uniquely unexpected and disruptive, the COVID-19 pandemic has similarities with other disrupters of paid work, such as childbirth, family, and health emergencies. Such events especially affect women, who are seen as traditional caregivers and perceived to have more time flexibility. This leads to greater economic losses compared to men, leaving women more vulnerable, further perpetuating gender inequities. Many women lack adequate support to deal with such events. Thus, building supportive structures for self-employed young women is an urgent imperative.

In Nigeria, there are ongoing programs sponsored by the government and other financial institutions targeted at women in business. An example is the *Women in Self-employment Program* domiciled in the Small and Medium Enterprises Development Agency of Nigeria (SMEDAN), which provides self-employed women with financial assistance, business training, mentorship, and access to markets [21]. Another example is the *Womenpreneur Pitch-a-ton* organized by Access Bank, which offers mini Master of Business Administration scholarships to beneficiaries alongside grants [22]. There have been testimonials on the positive effects of these programs, but the evidence for these remains anecdotal. The impact of these programs is not evaluated in the systematic way that trials are done, thus limiting the examination of their effectiveness and generalizability. In addition, the development and implementation of most of these programs lack the vital element of coproduction, thus limiting impact. Coproduction [23] is a collaborative, participatory approach where those who will ultimately benefit or be impacted by research or an intervention initiative are a part of its development process; research or the intervention development and implementation is conducted with them rather than on them. Participatory approaches are more likely to result in research or interventions that are shaped by the needs, values, and goals of the stakeholders, thereby increasing the uptake of the output. Beyond these instrumental gains, coproduction with those with lived experience is also an ethical and rights-based imperative. In sum, our Advancing Resiliency in Self-Employed Young Women in Nigeria (ARISE&WIN) study seeks to bridge these gaps in the lack of in-depth knowledge about the lives, stressors, and coping strategies of young self-employed women and the lack of coproduced and well-evaluated interventions to support their resilience and well-being.

### Research Aims

The project’s first aim is to conduct a gendered situational analysis of how the COVID-19 pandemic and other significant life events (childbirth, family, and health emergencies, etc) affect the paid and unpaid work and mental, physical, and social well-being of self-employed young women in Nigeria; their strategies for coping with such events; and how their experiences compare with those of self-employed young men. To achieve this aim, the project has the following objectives:

1. Explore through qualitative interviews, digital storytelling, and a scoping review self-employed young women’s and men’s experiences of the COVID-19 pandemic and life events that impact work and well-being
2. Quantitatively examine the gender-differentiated impact of the pandemic on the work and well-being of self-employed young people in Nigeria using longitudinal secondary data

The project’s second aim is to coproduce nationally scalable intervention packages in order to support self-employed young women in Nigeria to cope better with disruptions to their work and promote their well-being. The specific objectives under this aim are as follows:

1. Synthesize existing evidence on interventions for social protection, job quality, and well-being promotion for self-employed youth using a systematic review, policy analysis, as well as qualitative interviews with self-employed young women, members of their support network, and policy makers
2. Coproduce, using theory of change and co-design workshops, intervention packages to improve the resilience of self-employed young women in Nigeria against significant life events

The third aim of the project is to pilot and assess the feasibility, acceptability, cost, and potential effectiveness of the coproduced intervention packages on the resilience, job quality, and mental and physical well-being of self-employed young women, using a quasi-experimental study. The study focuses on young women aged between 15 and 35 years [24].

## Methods

### Study Setting

The project is being conducted in Oyo State in southwest Nigeria. Oyo State is a suitable site to capture a wide range of self-employed young workers’ experiences, given its large and diverse population, with migrants from across Nigeria, and the baseline data that the research team has from here. The study is being conducted in urban, periurban, and rural local government areas (LGAs) of Ibadan, the capital of Oyo State, because of the proximity to certain facilities of the Oyo State Government that will be used in the pilot of the coproduced intervention packages. Ibadan is the third most populous city in Nigeria and has about 4 million inhabitants [25]. The main economic activities carried out by the inhabitants are agriculture, trade, public service employment, handicrafts, and factory work [26]. Ibadan is a highly commercial city, with the presence of markets and businesses all over the metropolitan area. Many young people in the state are engaged in self-employment across sectors, such as garment making, shoemaking, hairdressing, building construction, catering, and food vending. They also work as graphic designers, printers, electricians, auto mechanics, etc.

### Study Design

This is a multistage, multimethod research project. This project incorporates integrated knowledge mobilization strategies right from the development of the research aims through its implementation, evaluation, and dissemination to ensure that it is relevant to the target population of self-employed young women as well as feasible for and scalable by policy actors [27]. Self-employed young women were involved in the development of the research proposal and have been playing an integral part throughout the project, bringing their perspectives and experiences. Policy makers and decision makers were also involved in proposal development and have been playing an important role in the execution of this project to enable the integration of its findings into Oyo state policy as well as policies for other states in Nigeria.

### Data Collection Strategies

#### Aim 1: Situational Analysis

##### Overview

The aim of the situational analysis component of the project is to examine the impact of the COVID-19 pandemic and other significant life events on the work and well-being of self-employed young women compared to men as well as their coping strategies for dealing with such events. Furthermore, we are examining cultural, social, psychological, and physical factors that might put self-employed young women at a disadvantage compared to self-employed young men. To develop a comprehensive understanding, semistructured interviews, quantitative secondary data analysis, a scoping review, and digital storytelling will be conducted. The findings from this situational analysis will inform the coproduction of intervention packages to improve the resilience of self-employed young women in Nigeria in the face of significant disruptive life events.

##### Semistructured Interviews

###### Overview

Semistructured interviews will explore and help compare and contrast self-employed young women’s and men’s experiences during the pandemic and other events that disrupted their businesses. These interviews also explore respondents’ strategies for coping with disruptive events, including leveraging social networks. The interview guide is provided in Multimedia Appendix 1.

###### Sampling

Self-employed young women and men will be sampled from urban, periurban, and rural areas in the Ibadan metropolitan area using both purposive and snowball sampling techniques until theoretical saturation is reached. Self-employed young women and men will be sampled from various types of businesses, including services, commerce, craftsmanship, primary production, and manufacturing, and self-employed youth with various levels of educational attainment will also be included. They will be recruited through various trade and business-related associations and other economic or social networks. Self-employed youth who are particularly vulnerable, including those with mental and physical disabilities, and adolescents will be purposively sampled. The total number of planned participants for the semistructured interviews is 60, roughly evenly divided between women and men based on an estimate of the number of participants we think we need to reach theoretical saturation (informed by previous qualitative studies of similar topics) [20]. Interviews will be audio recorded and transcribed verbatim. Transcripts will be analyzed as the study is ongoing, and the team will meet and decide whether the sample size needs to be reduced if theoretical saturation is achieved earlier (ie, the same themes repeat across interviews) or increased if it is not achieved (ie, new themes are still emerging).

###### Analysis

The audio-recorded interviews will be transcribed. Interviews conducted in Yoruba will be transcribed in Yoruba and translated into English, with caution taken to ensure that any nuances in communication are not lost. A practical thematic analysis will be conducted using ATLAS.ti software (version 24; Lumivero). Practical thematic analysis reframes the reflexive thematic analysis by Braun and Clarke [28] into 3 steps: reading, coding, and theming [29]. All members of the qualitative coding team will read through all the transcripts and code a few of the transcripts to develop a codebook. Then, each transcript will be coded independently by 2 members of the team, and the codebook will continue to be updated. There will be regular meetings to discuss emerging codes, and team members will be encouraged to regularly write reflective memos on their positionality as well as emerging findings. After the transcripts are coded, themes will be drawn out, and a thematic analysis session will be held, bringing all members of the qualitative analysis team together to decide on the final themes. To ensure the trustworthiness (credibility, dependability, transferability, and confirmability) of this qualitative research undertaking, researchers involved in collecting and analyzing the data will be encouraged to be reflexive by regularly writing field notes during data collection and memos during analysis. Insights from the field notes and memos will be incorporated while writing up to contextualize our findings. Reflecting our coproduction approach, self-employed young women are involved in data collection and analysis. To ensure the confirmability of our findings, the coding team will hold regular meetings to review and discuss emerging codes and themes. We will also do a member check-in with some of our participants so they can review the accuracy of our findings.

####### Quantitative Secondary Data Analysis

The Nigerian General Household Survey (GHS), Panel 2018-2019, and the COVID-19 National Longitudinal Phone Survey (COVID-19 NLPS), which covered all 36 states of Nigeria and the Federal Capital Territory, are being analyzed to assess the impact of the pandemic on the work and well-being of self-employed youth [30,31]. The COVID-19 NLPS sampling frame was derived from the GHS, so data from both surveys can be merged. The GHS panel collected individual and household-level data from 4976 households and the COVID-19 NLPS from 1950 households. The GHS panel data, collected before the COVID-19 pandemic, are serving as baseline data. From 2020 to 2021, 12 NLPS rounds were conducted, which can be compared with the GHS to understand the impacts of COVID-19. Both surveys have questions on health, employment, income status or changes, and coping with shocks. We are analyzing how factors such as sociodemographic characteristics, well-being, unpaid work, and access to banking and technology facilities affect the exit from self-employment to unemployment. The analysis will involve descriptive statistics and mixed models for longitudinal data. The work and health outcomes of self-employed young men and women are being compared, and self-employed youth are being compared with other age groups. Findings from these analyses will yield a comprehensive picture of the impact of the pandemic on self-employed young people.

##### Scoping Review

###### Overview

We are conducting a scoping review on the gendered impact of the COVID-19 pandemic and other significant life events and experiences on the job quality and well-being of self-employed workers in LMIC. The scoping review protocol has been registered with the Open Science Framework [32].

Thus far, no scoping review has been conducted to examine the impact of the COVID-19 pandemic and other significant life events on self-employed workers. Our scoping review will answer the following research questions:

1. What are the common significant life events and experiences that self-employed workers in LMIC face?
2. How do these significant life events and experiences, including the COVID-19 pandemic, affect the job quality and well-being of self-employed workers in LMIC, and how do they cope?
3. Are there differences between self-employed women versus men in terms of the occurrence and impact of significant life events and experiences and COVID-19 on their well-being and job quality and how they cope?

###### Eligibility Criteria

Studies where self-employed workers are the population of interest will be included. We will include studies that examine the impact of the COVID-19 pandemic and significant life events on the job quality and well-being of self-employed workers. Studies conducted in LMIC, based on the World Bank’s classification, will be included. The review will examine both peer-reviewed and gray literature published between 2003 and 2023. No study designs will be excluded from this review. We will not place a language restriction on the studies included in the scoping review. Non-English studies, which can be translated using Google Translate or through the services of native speakers, will be included.

###### Information Sources

The following databases will be searched: Global Health, Education Resources Information Center, PsycINFO, Business Source Ultimate, EconLit, Scopus, Sociological Abstracts, (ASSIA), Social Services Abstracts, ABI/INFORM Collection, Sociology Database, Social Science Database, SciELO, MEDLINE, and Cochrane CENTRAL. We will also search the websites of the World Bank and the International Labour Organization for relevant reports and working papers.

###### Search Strategy

The search strategy will include key terms derived from the scoping review research questions, including “self-employed worker,” “entrepreneur,” “business owner,” “significant life event,” “COVID-19 Pandemic,” “job quality,” “quality of work,” “physical wellbeing,” “mental wellbeing,” “social wellbeing,” and “coping.” The Cochrane Effective Practice and Organisation of Care filter for LMIC will also be used [33]. A sample search strategy for the MEDLINE (Ovid) database is included in Multimedia Appendix 2.

###### Selection of Sources of Evidence

Studies identified through the search will be imported to Covidence (Veritas Health Innovation Ltd). Covidence is a web-based collaboration software platform that streamlines the production of systematic and other literature reviews [34]. Two independent reviewers will screen the title and abstract to select eligible studies. After titles and abstracts are screened, we will conduct a full-text screening of the studies. Any screening-related conflicts will be resolved by a third reviewer.

###### Data Charting Process

Data charting will be done independently by 2 reviewers. Any disagreements between 2 reviewers will be resolved by referring to a third reviewer for adjudication. We will hold a calibration exercise where the data charting form will be piloted by members of the team to ensure that it captures all relevant data.

###### Data Items

We will extract data on the dimensions specified in Table 1.

**Table 1:** Data items.

Dimensions	Details
General information	Author, year, country of study, and study setting (urban or rural)
Study characteristics	Objectives of the study and study methodology
Study population	Sampling strategy, sample size, and sociodemographic characteristics of participants
Significant life events and experiences	Various kinds of significant life events and experiences that self-employed workers face
Impact of significant life events	Impact of significant life events on job quality and well-being
Impact of the COVID-19 pandemic	Impact of the COVID-19 pandemic on job quality and well-being
Coping	Coping strategies of self-employed workers to deal with job quality and well-being
Gender differences	Gender differences in the impact of significant life events and the COVID-19 pandemic on job quality and well-being as well as coping strategies of self-employed workers

###### Synthesis of Results

We will present a narrative synthesis of the extracted data on the impact of significant life events and experiences, including the COVID-19 pandemic, on job quality and well-being of self-employed workers in LMICs. The findings will be thematically analyzed and organized as advised by Levac et al [35]. A summary table will be presented showing the extracted data items (article characteristics, study characteristics, and key findings). Any relevant descriptive statistics obtained during data extraction will be used to supplement the narrative synthesis. The PRISMA-ScR (Preferred Reporting Items for Systematic Reviews and Meta-Analyses Extension for Scoping Reviews) checklist is in Multimedia Appendix 3.

####### Digital Storytelling

This arts-based participatory method combines storytelling and technology to produce a film telling a personal or community story [36]. Digital stories are 3- to 5-minute videos that showcase a person’s story. Digital storytelling will capture the experiences of self-employed young women and how they coped with the pandemic and other significant disruptive life events. In total, 10 women will be recruited to participate in the digital storytelling. The digital storytelling workshop manuals will be coproduced, and self-employed young women will be guided through the process of crafting narratives and producing their digital storytelling videos in a series of workshops. During these workshops, self-employed young women will have the opportunity to brainstorm ideas and choose their own individual stories they want to share via this medium. The focus of the stories will be on reflecting on their significant life experiences and how they overcame them. They will be supported to develop their story idea into a storyboard detailing how they will incorporate music, pictures, and other elements in the video. They will also be provided with technical support to convert these storyboards into videos capturing their experiences. After the digital storytelling videos have been completed, we will host a film screening of the digital stories, which will include discussion sessions involving the self-employed women whose stories are being told as well as other relevant stakeholders, such as other self-employed young women and men, policy makers, researchers, and the public. These discussion sessions will provide a forum for self-employed women to talk about their experiences developing digital stories and for other stakeholders to reflect on the thoughts and feelings after listening to or watching these videos. To get more information about the film screening attendees’ perceptions of the stories shared for those who might not have been able to talk in the discussion sessions, a short web-based and phone survey will be administered to attendees after the film screening. A narrative analysis of the digital stories will be conducted to identify motifs emerging from the digital stories. The discussion sessions and survey will also be thematically analyzed to provide more insights into the impact of significant life events on self-employed young women and the community. The digital stories will be disseminated through web-based forums. The data from the digital storytelling process will shed light on the experiences of self-employed young women and the role stories can play in promoting resilience.

#### Aim 2: Coproduction of the Intervention Packages

##### Overview

The study’s second component is the coproduction of intervention packages to improve the resilience of self-employed young women. The intervention packages are being informed by synthesizing available evidence from the situational analysis phase of the project and stakeholders’ inputs. A theory of change approach will guide the coproduction of the intervention packages. This will facilitate the theory-driven design and evaluation of our proposed interventions and ensure their effectiveness and scalability [37]. The following methods will be used to coproduce the interventions: a systematic review, focus group discussions (FGDs) with self-employed young women and members of their support network and semistructured interviews with policy makers, policy analysis, and theory of change and coproduction workshops. Findings from the semistructured interviews with self-employed young men and women, quantitative secondary data analysis, digital storytelling, scoping review, systematic review, and qualitative interviews with members of the support network of self-employed young women and policy makers will be presented at the theory of change workshops. This will help inform the codevelopment of the theoretical map that will guide the choice and delivery of interventions on the project. In addition, the findings from the situational analysis and coproduction phases will inform the components of the intervention that will be developed and fleshed out in coproduction workshops.

##### Systematic Review

###### Overview

The systematic review will examine which interventions are effective in improving the job quality, well-being, and coping strategies of self-employed youth. The systematic review protocol titled “Protocol of a Systematic Review of Policies, Programs, and Interventions Designed to Improve the Job Quality and Well-being of Self-Employed Workers” has been registered on PROSPERO [38]. To improve the resilience of self-employed workers to withstand significant life events, it is important to identify which interventions will be most effective in improving their job quality and well-being. Furthermore, it is important to know to what extent self-employed workers were involved in the development of these interventions because coproduction is crucial in ensuring an intervention is relevant to its target population. The systematic review answers the following questions:

1. Which interventions are effective for improving the job quality, well-being, and coping strategies of self-employed workers? Are there gendered differences in the impact of these interventions?
2. To what extent have interventions that have been tested to improve the job quality, well-being, or coping strategies of self-employed workers included them as co-designers or taken gender into consideration?

The review will be reported in line with PRISMA (Preferred Reporting Items for Systematic Reviews and Meta-Analyses) guidelines [39]. The PRISMA-P (Preferred Reporting Items for Systematic Reviews and Meta-Analyses Protocols) checklist is included in Multimedia Appendix 5.

###### Eligibility Criteria

The participants of interest in this study are self-employed workers. Studies that report on interventions, programs, training packages, policies, and projects that aim to improve the job quality and resilience or well-being of self-employed workers will be included. Studies that have comparisons (control groups of individuals that did not have any intervention or those that had a less intensive or a different type of intervention) will be selected. Studies will be eligible if they include the primary outcomes of interest (well-being, job quality, and coping strategies) or secondary outcomes of interest (business performance, productivity, level of formality of their enterprises, and resilience). The review will consider the following study designs: randomized controlled trials (RCTs; including individual RCTs, cluster RCTs, and stepped-wedge designs), cohort studies, case-control studies, controlled before-and-after studies, interrupted time series studies, and controlled trials. The review will also include studies that use difference-in-difference estimation, synthetic control group methods, studies based on covariate matching, propensity-score–based methods, doubly robust methods, regression adjustment, regression discontinuity designs, and instrumental variable estimation. Systematic reviews, meta-analyses, and qualitative studies will be excluded. The review will include both peer-reviewed and gray literature published after January 1, 2023, until the date of the search. We will consider studies published anywhere across the globe. There is no language restriction on the studies that will be included in the systematic review.

###### Information Sources

The following databases will be searched: Global Health, Education Resources Information Center, PsycINFO, Business Source Ultimate, EconLit, Scopus, Sociological Abstracts, ASSIA, Social Services Abstracts, ABI/INFORM Collection, Sociology Database, Social Science Database, SciELO, MEDLINE, and Cochrane CENTRAL. The World Bank and International Labour Organization websites will also be searched.

###### Search Strategy

The search strategy is derived from the key terms in the research questions, including “self-employed worker,” “entrepreneur,” “intervention,” “program,” “policy,” “co-produced program,” “co-designed program,” “job quality,” “quality of work,” “wellbeing,” and “coping” alongside their synonyms. The search strategy will be tailored to the unique command language of each of the included databases. A sample search strategy for the MEDLINE (Ovid) database is included in Multimedia Appendix 4.

###### Selection Process

We will use Covidence, a web-based systematic review software, to manage the article selection and extraction process. Two reviewers will conduct title and abstract screening as well as full-text screening independently. Any conflicts arising during the abstract and full-text screening process will be resolved by a third reviewer who is an expert in the field.

###### Data Collection Process

Two reviewers will extract data from each eligible study. The information will be extracted from the eligible studies with the aid of Covidence and ATLAS.ti.

###### Data Items

The information presented in Textbox 1 will be extracted from each study.

**Data items.**

- Participants: sample size, size of intervention arms, and demographic information (age, gender, and employment)
- Interventions: type of intervention, length of intervention, content of intervention, implementation context, and consideration of gender in the design and development of intervention
- Outcomes: primary outcomes of job quality, physical well-being, mental well-being, and social well-being as well as secondary outcomes of business performance, level of formality of the enterprises, productivity, resilience, coping, and their follow-up time points
- Methods: study design, follow-up time points, study location, study time frame, and the use of coproduction involving self-employed workers
- Results: effect sizes (mean differences and risk ratios), CIs, and *P* values

###### Study Risk of Bias Assessment

The risk of bias will be assessed using the revised Cochrane Risk of Bias tool for randomized trials [40] and Risk of Bias in Nonrandomized Studies of Interventions [41] at the outcome level. If there are sufficient studies for a meta-analysis, sensitivity tests will be conducted to examine the risk of bias judgments and exclude studies of poor quality.

###### Synthesis Methods

We will present a narrative synthesis of the findings to describe study participants, study context, study design, intervention components, and outcomes of the included studies. This information will also be presented in tabular form for each study. If there are sufficient studies with consistent designs and outcomes, a meta-analysis will also be conducted using R software (R Foundation for Statistical Computing). Sensitivity analyses will be used to exclude poor-quality studies from the meta-analysis. Subgroup analyses will also be conducted based on the following characteristics: (1) age of the self-employed workers undergoing the intervention and (2) gender.

###### Reporting Bias Assessment

A thorough assessment of selective nonreporting or underreporting of results in the studies included in the meta-analysis (if carried out) will be used to assess the risk of bias due to missing results.

###### Certainty Assessment

The Grading of Recommendations Assessment, Development and Evaluation approach will be used to assess the certainty in the body of evidence that will be generated through this systematic review. This review will yield insights that will be helpful in developing the intervention packages for this project.

####### FGDs and Semistructured Interviews

Interventions from the literature cannot be applied to self-employed young women in Oyo State without ensuring their feasibility and cultural relevance. Focus groups will be conducted with self-employed young women and members from their support network (eg, husbands and parents) to determine appropriate elements in a coproduced intervention. Interviews will be conducted with policy makers involved in the development or implementation of policies and programs that are of relevance to self-employed young women, such as policy makers in the Federal Ministry of Women Affairs; the Federal Ministry of Youth Development; the Federal Ministry of Industry, Trade and Investment; and the Development Agenda for Western Nigeria Commission to gain their perspectives on appropriateness, fundability, and alignment with policy priorities for potential interventions, as it was considered more acceptable and feasible to conduct interviews (and not focus groups) with policy makers. Sample sizes will be based on theoretical saturation considerations, but we anticipate the participation of 28 self-employed young women and 21 members of their support network in the FGDs. We also anticipate the participation of 8 policy makers in the semistructured interviews. The sample size estimations are informed by previous qualitative studies on similar topics [42]. The audio recordings of the FGDs and semistructured interviews will be transcribed. The Yoruba transcripts will be translated into English. We will analyze the transcripts using reflexive thematic analysis with the aid of ATLAS.ti to identify what self-employed young women, members of their support networks, and policy makers consider to be contextually appropriate interventions. There are Yoruba-speaking members on the team to ensure that the analysis is contextual. Similar procedures will be followed as detailed in the analysis of semistructured interviews under the Aim 1: Situational Analysis section to ensure the trustworthiness (credibility, transferability, confirmability, and dependability) of the analysis process.

####### Policy Analysis

The policy analysis focuses on analyzing policies of relevance to self-employed youth in Nigeria to inform the development of contextually appropriate interventions. As part of the policy analysis, we will identify policies relevant to self-employed youth in Nigeria. We will also evaluate the effectiveness of policy strategies and policy implementation in supporting self-employed women in Nigeria and enhancing their resilience against disruptive life events. To accomplish this objective, we will thematically analyze policy documents and secondary literature that evaluate the effectiveness of identified policies. The analysis of these policies has been informing the identification of policy goals for the project as well as the mapping of relevant stakeholders to achieve the policy goals.

####### Theory of Change and Coproduction Workshops

**Figure 1:**
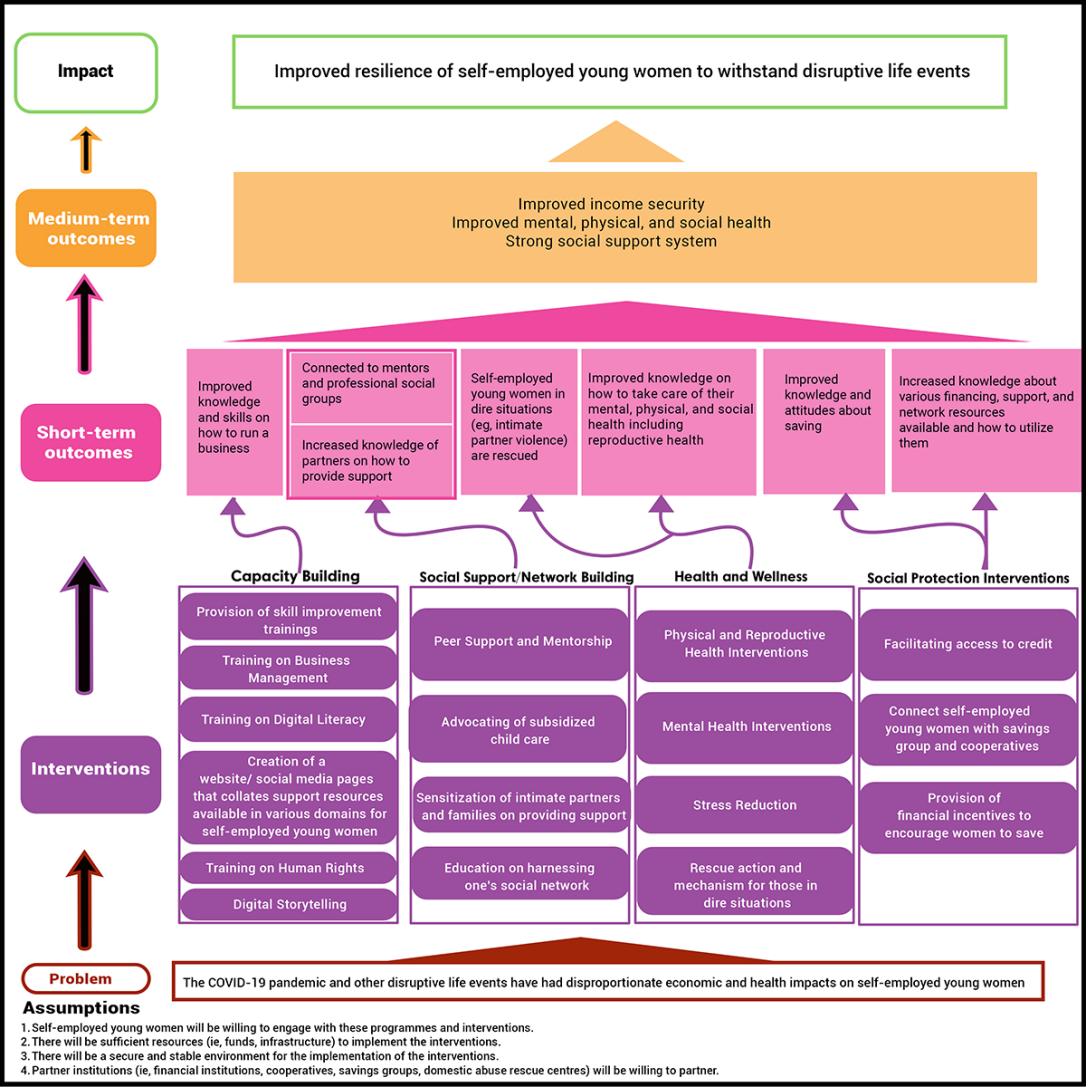
First Version of Theory of Change Map.

While developing the protocol for this study, theory of change workshops were held in June 2022 involving the entire team, comprising self-employed young women, policy makers, and researchers, to develop a theoretical framework to guide the choice of interventions for this study. The theory of change map developed through these workshops can be found in Figure 1. Following this, a few more theory of change workshops have been held, which have led to the refinement of this map. The overall impact detailed in the theory of change map in Figure 1 is “improved resilience of self-employed young women to withstand disruptive life events.” This theory of change map details 4 broad categories of potential interventions. The intervention categories are capacity building, social protection and network building, health and wellness, and social protection interventions. The theory of change map also includes short- and long-term outcomes.

Coproduction workshops will also be held, bringing together self-employed young women, policy makers, and researchers to develop the content of the intervention packages. At the coproduction workshops, participants will brainstorm the contents of the intervention packages as well as develop, review, and refine intervention materials.

#### Aim 3: Pilot Trial of the Intervention Packages

##### Overview

The aim of this phase of the project is to pilot the coproduced intervention packages developed in phase 2 of the project to assess their feasibility, acceptability, cost, and potential effectiveness. This phase will also assess the factors that facilitated and hindered the implementation of the piloted intervention packages and what needs to be learned before scaling up.

The pilot of the ARISE&WIN interventions has been planned as a quasi-experimental study with a prepost design. The design was chosen because there was unanimity around it being feasible while allowing us to draw conclusions about the potential effectiveness of various interventions. The study will consist of 3 arms. One of the intervention groups will receive the capacity building intervention package. The second intervention group will receive the social protection intervention package in addition to the capacity building intervention package. The capacity building and the social protection interventions will be finalized during the coproduction process. The third group will be a control group serving as a comparison group, and participants will not receive any intervention. The pilot will be conducted in the LGAs of Greater Ibadan, where situational analysis has been conducted. The LGAs will be stratified into urban, periurban, and rural LGAs and randomly assigned to 1 of the 2 intervention groups or the control group. On the basis of recommendations from literature [43,44] on pilot trials, a sample size of 120 participants would be large enough to assess feasibility and other outcomes of interest in the pilot study. To account for possible attrition, the sample size would be rounded up to 150 in total, with 50 participants per arm of the trial. Frequency matching will be conducted to ensure that across the intervention and control LGAs, the participants will have similar characteristics based on age and their sector of occupation. Participants will be recruited through advertisements on social media platforms where self-employed young women are active. Announcements will also be made at business association meetings to identify eligible participants. Participants will also be recruited through partner organizations (SMEDAN and the Centre for Support of Women in Unpaid and Informal Employment in Nigeria) that work with self-employed young women.

All groups will participate in a preintervention survey. Then, the interventions will be delivered over a period of 4 months. Follow-up surveys will be conducted at 6 weeks at the end of the capacity building intervention and months 4, 6, and 12. As part of a process evaluation, in-depth exit interviews will be conducted with approximately 20 participants to gain deeper insights into their perceptions about the interventions they receive. In addition, a survey will be administered to intervention facilitators while a subset of them will participate in in-depth interviews.

##### Feasibility

The feasibility of recruitment will be measured by the percentage of the sample size that is recruited. To measure the retention rate of participants, the attendance of the participants in the intervention training sessions and their level of compliance with intervention guidelines will be recorded. Furthermore, facilitators and participants in the intervention will be asked questions about the feasibility of scaling the intervention and conducting a similar but larger-scale trial in both the survey questionnaires and in-depth interviews that will be administered at the end of intervention delivery.

##### Acceptability

To assess the acceptability of the interventions to the participants, they will be asked about how helpful they found the intervention, whether they liked the way the intervention was delivered, and their thoughts about the data collection instruments and the frequency of administration. The acceptability of the RCT design will also be assessed.

##### Cost

Comprehensive records of all costs incurred in implementing the intervention will be kept, including the costs of recruiting the participants and other intervention implementation costs, such as facilitator and participant compensation, training materials, equipment and venue renting, catering costs, transportation, and childcare costs of the participants. This will be used to calculate total costs as well as per-unit costs for participants.

##### Outcomes

The primary outcome of this study is well-being (financial, mental, and physical). Financial well-being will be measured using the Consumer Financial Bureau Financial Well-Being Scale [45]. Mental and physical well-being will be measured using the 9-item Patient Health Questionnaire [46], the 12-item General Health Questionnaire [47], as well as the 12-item Short-Form Health Survey [48].

The secondary outcomes that will be measured are earnings, savings, perceived social support, as well as access to health care. Earnings will be measured by asking questions about participants’ revenues, costs, and profits from their sales. Savings will be measured by asking about the various persons, institutions, or organizations with which they save; how frequently they save; and how much they save each time they do. Social support will be measured using the Multidimensional Scale of Perceived Social Support [49] and the Self-Employment Social Support Scale [20].

In order to understand the pathways through which the interventions could affect the outcomes, questions will also be asked, which evaluate the knowledge, attitude, and practices of the self-employed young women who participate in the intervention in the various domains of the intervention, including business management; the use of digital technologies; the management of their personal and business finances; the use of resources available to self-employed young women in Oyo State; and caring for their mental, physical, and social health. Questions will also be asked to find out the number of business associations and other social groups that self-employed young women belong to, whether they have been rescued from any dire situations (eg, domestic violence), and how many of them are enrolled in health insurance. Questions will also be asked to members of the support network of self-employed young women who participate in the intervention to assess changes in their knowledge, attitudes, and practices toward providing support to the self-employed young women in their lives. Furthermore, the pre- and postintervention survey will include 3 rated items and an open-ended question about the impact of the last experienced significant life event on participants’ ability to meet their needs and on their well-being and work.

##### Analysis

Participants’ background characteristics will be summarized using frequencies and percentages for categorical variables, such as education, religion, marital status, etc. Means and SDs will be computed for numerical variables, such as age and income. The comparison of study outcomes across the 3 groups will be done using chi-square tests and 1-way ANOVA, as appropriate, at *P*<.05. Pairwise comparisons will also be done to compare the 2 intervention groups with the control group. Bonferroni adjustment will be made to correct for error rates that may be introduced by multiple comparisons. If necessary, generalized linear models will be fitted to adjust for confounders and pertinent variables that exhibit baseline differences between the study groups (eg, education status). Descriptive statistics and multivariate regressions will be adopted to analyze the Likert-scale questions in the survey on the feasibility and acceptability of the interventions to the participants and facilitators. All qualitative data in the study will be analyzed using the reflexive thematic analysis method.

### Ethical Considerations

The project will follow international research standards and be guided by the Principles for Global Health Research by the Canadian Coalition for Global Health Research. Ethics approval has been obtained from the Oyo State Ministry of Health Research Ethics Committee (reference number: NHREC/OYOSHRIEC/10/11/22), University of Ibadan and University College Hospital Ethics Committee (reference numbers: UI/EC/23/0256 and UI/EC/24/0809), and McGill University Ethics Committee (reference number: A11-B42-23A). Written informed consent or assent will be obtained from all participants before their enrollment into the study. Participants aged <18 years will be required to provide assent, while informed consent will be obtained from their parents. No names or any other forms of identification will be used in publications or reports resulting from the study without the permission of the participants. In keeping with recommendations for digital storytelling, participants will have the choice of identifying themselves and sharing their testimony. The consent and assent forms, interview guides, and questionnaires have been translated to Yoruba, the local language of the study location, using World Health Organization–recommended translation and back-translation procedures. This has been done to ensure that participants understand the study procedure fully and make an informed decision about whether to participate or not. Participants will be compensated monetarily for their time participating in this study as well as their transportation costs. This compensation will range from 2000 Nigerian naira to 10,000 Nigerian naira depending on the number of hours of participation. The conversion rate is 1 naira=US $0.00062).

### Data Management

All data will be digitally stored on the secure servers of the University of Ibadan and McGill University. Data will be stored in nonproprietary file formats wherever possible. All data will be anonymized and delinked from identifying information to ensure privacy and confidentiality. Knowledge translation materials will be openly available and uploaded to the International Development Research Centre repository. Scientific publications will be available open access.

### Integrated Knowledge Translation

Table 2 lists target groups for the research project and the knowledge mobilization strategies that will be used to engage them.

Knowledge mobilization strategies have been continuously further refined based on the input of other stakeholders. The principles of coproduction and equity have been guiding knowledge mobilization activities (eg, plain language summaries will be created for self-employed young women and tailored summaries for policy makers and associations). Our project steering committee has also been advising us on knowledge translation for scale and impact. In summary, our knowledge translation will guide Oyo State, Nigerian, and global efforts to enhance the resilience, health, and well-being of self-employed young women against significant disruptive life events.

**Table 2:** Integrated knowledge mobilization strategies.

Knowledge users	Strategies
Self-employed young women	Involvement of self-employed young women in the development, implementation, and dissemination of the findings of the project Presentation of findings by “experts by experience” to associations and groups that the self-employed young women belong to Dissemination of videos created through digital storytelling
State, regional, and national policy and decision makers	Involvement of state and regional policy makers in the design and implementation of the project Targeted dissemination of policy briefs, intervention guidelines, digital storytelling videos, and peer-reviewed articles to policy makers Meetings and seminars with policy makers to inform them of the findings of the project and advocate for the scale-up of coproduced interventions
Scientific community and researchers	Peer-reviewed publications Conference presentations
General public	Dissemination of the findings of the project through press releases, social media platforms, and web content, as well as open-access publications

## Results

The recruitment of participants for the situational analysis phase of the project began in May 2023 and ended in January 2024. The recruitment of participants for the pilot study began in November 2024 and will be completed in January 2025. Data collection will be completed in November 2025. Table 3 gives details on the status of the various components of the study.

**Table 3:** Status of various components of the Advancing Resiliency in Self-Employed Young Women in Nigeria (ARISE&WIN) study.

Activity	Status
Semistructured interviews with self-employed young women and men	Interviews have been conducted and are being analyzed.
Quantitative secondary data analysis	Analysis of secondary data is ongoing.
Scoping review	We are currently conducting full-text screening.
Digital storytelling	Digital stories have been created and disseminated. We are writing our analysis of these stories and their reception.
Systematic review	We are extracting data and conducting risk of bias assessments for selected studies.
Focus group discussions with self-employed young women and members of their support network and key informant interviews with policy makers	Interviews have been conducted and are being analyzed.
Policy analysis	Policies have been analyzed. We are writing the results.
Theory of Change and coproduction workshops	Theory of change and coproduction workshops have been conducted.
Pilot trial of the intervention packages	The intervention packages are currently being delivered. Data collection will be completed in November 2025.

## Discussion

This is a gender-transformative project that will provide a greater understanding of the impact of the COVID-19 pandemic and other significant disruptive life events on the working lives and well-being of self-employed young women in Nigeria and the norms, attitudes, and structures that perpetuate gender inequity. Through a collaboration between the researchers, self-employed young women, policy makers, and other relevant stakeholders, the project will result in suitable and contextually appropriate gender-transformative interventions to enable young women to better cope with events that disrupt their work and well-being and improve their income security; their mental, physical, and social health; and their social support system. The interventions that will be developed will consider the perspectives and needs of women who are particularly marginalized, including those with mental and physical disabilities and those with little or no formal education. These interventions will differ from previous interventions for self-employed young women in this context because they will be coproduced, and intentional attention will be paid to gender dynamics in their design. The results of the pilot study will be used to ascertain the feasibility, acceptability, cost, and potential effectiveness of the coproduced intervention packages. The results of the pilot study will be used to refine the intervention packages in preparation for a large-scale efficacy trial.

Research and policy-related findings will be disseminated through peer-reviewed articles, policy briefs, intervention guidelines and manuals, press releases, social media, and web content. Multistakeholder meetings involving self-employed young women, government officials, impact investors, researchers, and other relevant individuals will be organized to disseminate and discuss the findings from the various stages of research over the course of the project. The partnership with Oyo State Ministry of Women Affairs and Social Inclusion, SMEDAN, and the Centre for Support of Women in Unpaid and Informal Employment in Nigeria in the development and rollout of this project will enhance the uptake and scale-up of the intervention. Our partnership with the Development Agenda for Western Nigeria Commission, an organization responsible for facilitating policy design and implementation for the 6 states in the southwest of Nigeria, will enable the scale-up of the policy findings from this project to other Nigerian states.

This project will build capacity among self-employed young women to coproduce and contribute to research and policy development. The project will also develop their capacity by improving their knowledge and skills on how to run a business, improve their knowledge on how to take care of their health, and enable them to harness existing networks to better support their businesses through disruptive events. The project will enhance the capacity of the research team and policy makers in coproduction so that research and policy have greater relevance to the lived realities of young, self-employed Nigerian women.

Ultimately, this project will help advance Sustainable Development Goal (SDG) 3 “to ensure healthy lives and promote well-being for all at all ages”; SDG 5 to “achieve gender equality and empower all women and girls”; and SDG 8 “to promote sustained, inclusive, and sustainable economic growth, full and productive employment, and decent work for all.” It will also advance SDG 1 “nobody will live in extreme poverty,” SDG 2 “end hunger and achieve food security,” and SDG 16 “promote peaceful and inclusive societies for sustainable development.”

## Appendix

Multimedia Appendix 1 Interview guides for semistructured interviews and focus group discussions.

Multimedia Appendix 2 Scoping review sample search strategy.

Multimedia Appendix 3 PRISMA-ScR (Preferred Reporting Items for Systematic Reviews and Meta-Analyses Extension for Scoping Reviews) checklist.

Multimedia Appendix 4 Systematic review sample search strategy.

Multimedia Appendix 5 PRISMA-P (Preferred Reporting Items for Systematic Reviews and Meta-Analyses Protocols) checklist.

Multimedia Appendix 6 Peer review report by the International Development Research Center (Canada).

## Abbreviations

ARISE&WIN: Advancing Resiliency in Self-Employed Young Women in Nigeria
COVID-19 NLPS: COVID-19 National Longitudinal Phone Survey
FGD: focus group discussion
GHS: Nigerian General Household Survey
LGA: local government area
LMIC: low- and middle-income countries
PRISMA: Preferred Reporting Items for Systematic Reviews and Meta-Analyses
RCT: randomized controlled trial
SDG: Sustainable Development Goal
SMEDAN: Small and Medium Enterprises Development Agency of Nigeria

## Data Availability

The datasets generated or analyzed during this study are available from the corresponding author on reasonable request.
