# Supplementary material for: Understanding the Gendered Impact of COVID-19 on Young Self-Employed Nigerian Women and Coproducing Interventions That Foster Better Systems and Well-Being: Protocol for a Multimethods Study
Source: JMIR Res Protoc. 2025 May 30;14:e69577. doi: 10.2196/69577 (PMC12166318; doi:10.2196/69577)
Supplement: Multimedia Appendix 1 [file resprot_v14i1e69577_app1.docx]

**Template for Semi-Structured Interview Schedule for Interviews with Self-Employed Young Women and Men**

*Introduce Yourself*

*Explain the purpose of the interview:* The purpose of this interview is to find out about how the COVID-19 pandemic and other significant life events have affected your work and family life.

*Confirm participant’s readiness, and seek written consent, explain that the interview will be recorded.*

**Background**

1. Tell me about yourself.
2. Tell me about your family.
3. What jobs do your parents/guardians and siblings do? If you are married, what job does your husband/wife/partner do?
4. When did you start working?
5. What jobs have you done since you started working?

*Probes*

a. Let’s start from your very first job.

b. Was this your very first job?

**Transition into Self-Employment**

1. I understand you are self-employed. Please tell me about your business.

*Follow-Up*

1. How did you become self-employed?
2. Why did you become self-employed?
3. How do you feel about running your business?

**Significant Life Events**

1. Can you tell me about any important life events or experiences you have had in your life? We are particularly interested in those events that happened after you started working.

*Probes*

1. **Can you tell me about any memorable events that you have had?**

(i) How about marriage?

(ii) How about childbirth?

1. **Can you tell me about any difficult/ traumatic events that you have had?**

(i) How about health emergencies?

(ii) How about disasters (e.g. fire, flood)?

(iii) How about a family emergency?

(iv) How about 419?

(v) How about indebtedness?

(vi) How about fear of failing?

1. How about the growth of the business?
2. Can you describe how these significant events or experiences impacted you or your life?

*Probes*

a. Can you describe how these significant events or experiences impacted your family?

b. Can you describe how these events or experiences affected your health or wellbeing?

- - 1. Physical Health
    2. Mental Health

iii. Social Health/ life with families and friends

c. Can you describe how these events or experiences affected your business?

d. Can you describe how these events or experiences affected your financial well-being?

1. How did you cope with the effects of these significant events or experiences? We are particularly interested in how you coped with those significant events that happened after you started working.

*Probes*

a. How did you cope with the effects of these significant events or experiences on your family life?

b. How did you cope with the effects of these significant events or experiences on your health?

1. Physical Health
2. Mental Health

iii. Social Health// life with families and friends

c. How did you cope with the effects of these significant events or experiences on your **business**? How did you cope with the effects of these significant events or experiences on your **financial well-being**?

**COVID-19 Pandemic**

1. Tell me about your experience during the COVID-19 pandemic.

*Probes*

a. How has the COVID-19 pandemic affected your family life?

b. How has the COVID-19 pandemic affected your health?

1. Physical Health
2. Mental Health

iii. Social Health/life with family and friends

c. How has the COVID-19 pandemic affected your business?

d. How has the COVID-19 pandemic affected your financial well-being?

1. How did you cope with the effects of the COVID-19 pandemic?

*Probes*

a. How did you cope with the effects of the COVID-19 pandemic on your family life?

b. How did you cope with the effects of the COVID-19 pandemic on your health?

i. Physical Health

ii. Mental Health

iii. Social Health/life with family and friends

c. How did you cope with the effects of the COVID-19 pandemic on your **business**? How did you cope with the effects of the COVID-19 pandemic on your **financial well-being**?

1. Did you receive assistance from any person or organization to cope with the effects of the COVID-19 pandemic on you, your business, your health or family?

**Family and Health Emergencies**

1. Please tell me about a time that you or a family member experienced ill health and could not work.

*Probes*

a. How did this affect you?

b. How did this affect your family life?

c. How did this affect your business?

d. Is there any other similar experience you would like to share with me?

1. How did you cope in these instance[s]?
2. How did you help your family member cope?

**Naira Re-Design Policy**

16. How did the naira re-design policy affect your business?

*Follow-up*

i. How were you able to cope with the effects of the naira re-design policy?

**Differences Between the Experiences of Self-Employed Young Men and Women**

17. How do you think the experiences of self-employed young men and women are similar and different? (Add if needed: We are particularly interested in their experiences with significant life events and how these impact their lives and work)

*Probes:*

- 1. Do you think that self-employed young women are at a disadvantage compared to self-employed young men or self-employed young men at a disadvantage compared to self-employed young women?
  2. Do you think that significant life events and experiences affect self-employed young men or self-employed young women more?

*Follow-up:* Why do you think so?

- 1. Are there any differences in the ways self-employed young men and women cope with difficult life events?
  2. Are there any differences in the resources or supports available to self-employed young men compared to self-employed young women?

**Wrap Up**

1. Is there anything else you would like to add to our conversation today?
2. Are there any ideas you have about how we can share the results of this study?
3. Are there any ideas on how we could use the results from this study?

**Template for Question Guide for Focus Group Discussions with Self-Employed Young Women on Appropriate Interventions for Self-Employed Young Women**

*Introduce Yourself*

*Explain the purpose of the meeting:* The purpose of this focus group discussion/key-informant interview is to brainstorm on what interventions should be put in place to support self-employed young women in Oyo State, Nigeria cope better with significant life events or experiences that disrupt their work and family life.

*Confirm participants’ readiness, seek written consent, explain that the discussion will be recorded.*

**Significant Life Events or Experiences**

1. What are some common significant life events or experiences that self-employed young women encounter?

*Probes*

a. What are common life events or experiences that could affect the work, health or family life of a self-employed young woman?

2. Can you tell me about any significant life events or experiences you have had? We are particularly interested in those events that happened after you started working.

*Probes*

- - - - 1. **Can you tell me about any memorable events that you have had?**

(i) How about marriage?

(ii) How about childbirth?

- 1. **Can you tell me about any difficult/ traumatic events that you have had?**

(i) How about health emergencies?

(ii) How about disasters (e.g. fire, flood)?

(iii) How about a family emergency?

(iv) How about 419?

(v) How about indebtedness?

(vi) How about fear of failing?

(vii) How about the COVID-19 pandemic?

- 1. How about the growth of the business?

3. Can you describe how these significant events or experiences impacted you?

*Probes*

a. Can you describe how these significant events or experiences impacted your family?

b. Can you describe how these events or experiences affected your health or wellbeing?

1. Physical Health
2. Mental Health
3. Social Health/life with family and friends

c. Can you describe how these events or experiences affected your business? Can you describe how these events or experiences affected your financial well-being?

4. How did you cope with the effects of these significant events or experiences?

*Probes*

a. How did you cope with the effects of these significant events or experiences?

b. How did you cope with the effects of these significant events or experiences on your health?

i. Physical Health

ii. Mental Health

iii. Social Health/life with family and friends

c. How did you cope with the effects of these significant events or experiences on your business? How did you cope with the effects of these significant events or experiences on your financial well-being?

**Interventions**

1. What support do you think would have helped you to cope better when you had these significant life events or experiences?

*Probes:*

a. What support would you have liked from your family?

b. What support would you have liked from friends?

c. What support would you have liked from your peers or professional groups?

d. What support would you have liked from the government?

e. Any other support?

6. Do you know of any policies or programmes that will help self-employed young women better cope with significant disruptive life events?

Follow-up

I. Could you please tell me about these policies or programmes?

II. Do you know about the Oyo State Health Insurance Agency (OYSHIA)?

7. We are specifically focused on self-employed young women. In what ways are the supports that self-employed women could benefit from similar or different from those that self-employed men could benefit from?

**Capacity Building Interventions**

8. Have you ever participated in any trainings or activities to improve your knowledge or skills on how to run your business?

*Probes*

a. Business Management Training (This is training on how to coordinate and organize business activities)

b. Digital Literacy Training (This is training on how to use computers, the internet and other technologies for your business activities)

c. Human Rights Training (This is training on how to uphold your rights and those of other people in your community)

d. Other things you would have liked to be trained in?

*Follow-up:*

I. What was your experience with this training?

9. What websites or social media pages (e.g Facebook, Twitter, Instagram) do you know that collate resources for self-employed young women?

*Follow-up/ Ibeere atẹ̀lẹ́:*

I. What is your view of these websites and social media pages?

10. How important do you think listening to the stories of how other self-employed young women have coped with significant disruptive life events or experiences is to help you be better prepared to face such events? Please explain further.

**Social Support/Network Building Interventions**

11. Have you ever participated in a mentorship programme (i.e. a programme where you are able to receive guidance and advice from role models) for self-employed young women?

*Follow-up:*

1. What were your experiences on the mentorship programme?

Probe:

a. Please describe who your mentor(s) were/are and your relationship with him/her/them.

1. What are your views on mentorship programmes for self-employed young women? Please explain.

*Probe*:

a. Please describe the benefits of mentorship for self-employed young women.

b. Are there any disadvantages of mentorship programmes for self-employed young women?

1. What are the barriers for self-employed young women to participate in mentorship programmes?

12. Are you aware of any peer support groups for self-employed young women?

*Follow-up:*

a. Are you part of any support groups for self-employed young women?

b. If yes, what have been your experiences in the support groups for self-employed young women?

c. Have any barriers stopped you from participating in a peer support group for self-employed young women?

1. What is your view on the importance of affordable day care for self-employed young women?

*Follow-up:*

I. Do you have access to affordable day care?

- 1. If yes, how does having affordable day care impact you?
  2. If you do not have affordable day care, how has it affected your family life and business?

14. Do you get any help/support from family members and members of your community?

*Probes:*

a. What is the nature of the support you receive?

b. Please tell us about any childcare support you receive.

*Follow-up:*

1. If yes, what support do you find helpful for coping with disruptive significant life events?
2. If not, what do you think is responsible for the lack of support you have from family members and members of your community?

**Health and Wellness Interventions**

15. Have you ever participated in any health or wellness training or interventions?

*Probes:*

a. Stress Reduction Education

b. Reproductive Health Education

c. Physical Health Education

d. Mental Health Education

*Follow-Up*

I. What health training and interventions do you think will help self-employed young women cope better with significant disruptive life events?

**Social Protection Interventions**

16. Do you have access to the financial credit that you need for your business?

*Follow-up*

1. If yes, how are you able to access financial credit for your business?
2. If not, what do you think are the barriers to you accessing financial credit for your business?

17. What programmes do you think can be put in place to help self-employed young women to save?

18. Do you have any other questions or comments about our discussion?

19. Are there any ideas you have about how we can share the results of this study?

20. Are there any ideas on how we could use the results from this study?

**Question Guide Template for Focus Group Discussions with Members of Support Network of Self-Employed Young Women & Key Informant Interviews with Policymakers on Appropriate Interventions for Self-Employed Young Women**

Introduce Yourself

*Explain the purpose of the meeting:* The purpose of this focus group discussion is to brainstorm on what interventions should be put in place to support self-employed young women in Oyo State, Nigeria cope better with significant life events that disrupt their work and family life.

*Confirm participant’s readiness, seek written consent, explain that the interview will be recorded.*

**Significant Life Events or Experiences**

1. What are some common significant life events or experiences that self-employed young women encounter?

*Probes*

a. What are common life events or experiences that could affect the work, health or family life of a self-employed young woman?

2. Can you describe how these significant events or experiences impact self-employed young women?

*Probes*

a. Can you describe how these significant events or experiences impact their families?

b. Can you describe how these events or experiences affect their health or wellbeing?

i. Physical Health

ii. Mental Health

iii. Social Health

c. Can you describe how these events or experiences affect their businesses? Can you describe how these events or experiences affect their financial well-being?

3. How do they cope with the effects of these significant events or experiences?

*Probes*

a. How do they cope with the effects of these significant events or experiences on their family life?

b. How do they cope with the effects of these significant events or experiences on their health?

i. Physical Health

ii. Mental Health

iii. Social Health

c. How did they cope with the effects of these significant events or experiences on their businesses? How did they cope with the effects of these significant events or experiences on their finances?

**Interventions**

5. What support do you think self-employed women need to cope better with significant life events or experiences?

*Probes*

a. How do you think family members can support them?

b. How do you think friends can support them?

c. How do you think their peers or professional groups can support them?

d. How do you think the government can support them?

6. Who are the people that you think should be in the support system of a self-employed young woman?

*Follow-up:*

I. Why so?

II. Any other people?

7. What support do you think members in the support system of a self-employed young women need such as yourself?

8. Do you know of any policies or programmes that will help self-employed young women better cope with significant disruptive life events?

Follow-up*:*

I. Could you please tell me about these policies or programmes?

II. Do you know about the Oyo State Health Insurance Agency (OYSHIA)?

9. We are specifically focused on self-employed young women. In what ways are the supports that self-employed women could benefit from similar or different from those that self-employed men could benefit from?

**Capacity Building Interventions**

10. Are you aware of programmes to improve the knowledge or skills of self-employed young women on how to run their businesses?

*Probes:*

a. Business Management Training (This is training on how to coordinate and organize business activities)

b. Digital Literacy Training (This is training on how to use computers, the internet and other technologies for business activities)

c. Human Rights Training (This is training on how to uphold one’s rights and those of other people in one’s community)

*Follow-up:*

I. What is your view of these programmes?

II. How important do you think these programmes are to improve the resilience of self-employed young women against significant disruptive life events or experiences? Please explain further.

11. What websites or social media pages (e.g Facebook, Twitter, Instagram) do you know that collate resources for self-employed young women?

*Follow-up*

I. What is your view of these websites and social media pages?

12. How important do you think it is for self-employed young women to listen to the stories of how other self-employed young women have coped with significant disruptive life events or experiences to be better prepared to face such events? Please explain further.

**Social Support/Network Building Interventions**

13. Do you know about any mentorship programmes (i.e. a programme where you are able to receive guidance and advice from role models) for self-employed young women?

*Follow-up*

I. Please tell us about these mentorship programmes.

II. What are your views on mentorship programmes for self-employed young women? Please explain.

*Probes/ Ìtọpinpin:*

a. Please describe the benefits of mentorship for self-employed young women.

b. Are there any disadvantages of mentorship programmes for self-employed young women?

III. What are the barriers for self-employed young women to participate in mentorship programmes?

14. Are you aware of any peer support group for self-employed young women?

*Follow-up*

I. What are your views on peer support groups for self-employed young women?

II. What barriers do you think stop self-employed young women from participating in peer support groups?

15. What is your view on the importance of affordable day care for self-employed young women? Please explain.

**Health and Wellness Interventions**

16. Do you know about any health or wellness training or interventions for self-employed young women?

*Probes:*

a. Stress Reduction Education

b. Reproductive Health Education

c. Physical Health Education

d. Mental Health Education

*Follow-Up*

I. What health training and interventions do you think will help self-employed young women cope better with significant disruptive life events?

**Social Protection Interventions**

17. What are the ways that self-employed young women access the financial credit they need for their businesses?

*Follow-Up*

I. What do you think are the barriers to self-employed young women accessing financial credit for their businesses?

II. What interventions can be put in place to help self-employed young women access financial credit?

18. What programmes do you think can be put in place to help self-employed young women save?

19. Do you have any other questions or comments about our discussion?

20. Are there any ideas you have about how we can share the results of this study?

21. Are there any ideas on how we could use the results from this study?
