# Supplementary material for: Understanding the Gendered Impact of COVID-19 on Young Self-Employed Nigerian Women and Coproducing Interventions That Foster Better Systems and Well-Being: Protocol for a Multimethods Study
Source: JMIR Res Protoc. 2025 May 30;14:e69577. doi: 10.2196/69577 (PMC12166318; doi:10.2196/69577)
Supplement: Multimedia Appendix 2 [file resprot_v14i1e69577_app2.docx]

**Scoping Review Sample Search Strategy from Medline via Ovid**

1. Entrepreneurship/
2. ("Self-employed workers" or "Freelance*" or "Entrepreneur*" or "Business owner*" or "CEO*" or "Chief Executive Officer*" or "self-employed").ti,ab,kf.
3. ("Self-employed wom*" or "Entrepreneur* wom*" or "Female Chief Executive Officer*" or "Female business owner" or "Female entrepreneur*" or "Business women" or "Female CEO*").ti,ab,kf.
4. ("self-employed men" or "Entrepreneur* men" or "Male entrepreneur*" or "Male Chief Executive Officer*" or "male business owner" or "Business men" or "Male CEO*").ti,ab,kf.
5. 1 or 2 or 3 or 4
6. ("Significant life event*" or "Significant life experience*" or "Memorable event*" or "Memorable experience*" or "Life course*" or "Important event*" or "Important experience*" or "Adverse event*" or "Landmark event*" or "Landmark experience*" or "Major life event*" or "Major life experience*" or "Adverse life event*" or "Adverse life experience*" or "Traumatic experience*" or "Traumatic event*" or "Stressful life event*" or "Stressful life experience*" or "Milestone event*" or "Milestone experience*" or "Life event*").ti,ab,kf.
7. COVID-19/
8. ("COVID-19" or "Covid-19*" or "COVID-19 Pandemic*" or "Covid-19 Pandemic*" or "Coronavirus*" or "SARS-CoV-2" or "COVID" or "2019-ncov" or "COV-19").ti,ab,kf.
9. 6 or 7 or 8
10. ("job quality" or "quality of work" or "income" or "earning*" or "profit*" or "work* hour*" or "Work-life balance" or "prospect*" or "Skill use and discretion" or "Level of skill*" or "Physical environment" or "Work hazard*" or "Work intensity" or "Job fulfilment" or "Job satisfaction" or "Decent work" or "Quality of Employment" or "Work Quality").ti,ab,kf.
11. Mental Health/ or "Quality of Life"/
12. ("Mental wellbeing" or "Mental well-being" or "Social wellbeing" or "Social well-being" or "Physical wellbeing" or "Physical well-being" or "Mental health" or "Physical health" or "Social connectedness" or "Social health" or "Happiness" or "Job satisfaction" or "Well-being" or "well being" or "wellbeing" or "Wellness" or "life satisfaction" or "Resilien*" or "Self-efficacy" or "Tenaci*" or "quality of life" or "positive affect").ti,ab,kf.
13. ("coping" or "Coping strateg*" or "coping skill*" or "Coping style*" or "Coping mechanism*" or "Coping behav*" or "cope").ti,ab,kf.
14. 10 or 11 or 12 or 13
15. (afghan* or africa* or albania* or algeria* or angola* or antigua* or barbuda* or argentin* or armenia* or aruba* or azerbaijan* or bahrain* or bangladesh* or bengal* or bangal* or barbados* or barbadian* or bajan or bajans or belarus* or belorus* or byelarus* or byelorus* or belize* or benin* or dahomey or bhutan* or bolivia* or bosnia* or herzegovin* or botswan* or batswan* or bechuanaland* or brazil* or brasil* or bulgaria* or burkina* or burkinese* or upper volta* or burundi* or urundi* or cabo verde* or cape verde* or cambodia* or kampuchea* or khmer* or cameroon* or cameroun* or ubangi shari* or chad* or chile* or china* or chinese or colombia* or comoro* or comore* or comorian* or mayotte* or congo* or zaire* or costa rica* or "cote d'ivoir*" or "cote d' ivoir*" or cote divoir* or cote d ivoir* or ivory coast* or ivorian* or croatia* or cuba or cuban or cubans or "cuba's" or cyprus* or cypriot* or czech* or djibouti* or french somaliland* or dominica* or ecuador* or egypt* or united arab republic* or el salvador* or salvadoran* or guinea* or equatoguinea* or eritrea* or estonia* or eswatini* or swaziland* or swazi* or swati* or ethiopia* or fiji* or gabon* or gabonese* or gabonaise* or gambia* or ((georgia or georgian or georgians) not (atlanta or california or florida)) or ghana* or gibraltar* or greece* or greek* or grecian* or grenada* or grenadian* or guam* or guatemala* or guyana* or guiana* or guyanese* or haiti* or hispaniola* or hondura* or hungary* or hungarian* or india* or indonesia* or iran* or iraq* or isle of man* or jamaica* or jordan* or kazakh* or kenya* or karabati* or korea* or kosovo* or kosova* or kyrgyz* or kirgiz* or kirghiz* or laos or lao or laotian* or latvia* or lebanon* or lebanese* or lesotho* or lesothan* or lesothonian* or basutoland* or mosotho* or basotho* or liberia* or libya* or jamahiriya* or lithuania* or macedonia* or madagasca* or malagasy* or malawi* or nyasaland* or malaysia* or malay* federation or maldives* or maldivian* or indian ocean or mali or malian* or "mali's" or malta or maltese* or "malta's" or micronesia* or marshallese* or kiribati* or marshall island* or nauru or nauran or nauruans or "naurian's" or mariana or marianas or palau or paluan* or tuvalu* or mauritania* or mauritan* or mauritius* or mexico* or mexican* or moldova* or moldovia* or mongol* or montenegr* or morocco* or moroccan* or ifni or mozambique* or mozambican* or myanmar* or burma* or burmese or namibia* or nepal* or new caledonia* or netherlands antill* or nicaragua* or niger* or oman or omani or omanis or "oman's" or pakistan* or palestin* or gaza* or west bank* or panama* or paraguay* or peru or peruvian* or "peru's" or philippine* or philipine* or phillipine* or phillippine* or filipino* or filipina* or poland* or polish or pole or poles or portugal* or portuguese or puerto ric* or romania* or russia* or ussr* or soviet* or rwanda* or rwandese or ruanda* or ruandese or samoa* or navigator island* or pacific island* or polynesia* or "sao tome and principe*" or sao tomean* or santomean* or saudi arabia* or saudi or saudis or senegal* or serbia* or seychell* or sierra leone* or slovak* or sloven* or melanesia* or solomon island* or norfolk island* or somali* or sri lanka* or ceylon* or "saint kitts and nevis*" or "st kitts and nevis*" or kittian* or nevisian* or saint lucia* or st lucia* or saint vincent* or st vincent* or vincentian* or grenadine* or sudan* or surinam* or syria* or tajik* or tadjik* or tadzhik* or tanzania* or tanganyika* or thai* or timor leste* or east timor* or timorese* or togo or togoles* or "togo's" or tonga* or trinidad* or tobago* or tunisia* or turkiy* or turkey* or turk or turks or turkish or turkmen* or uganda* or ukrain* or uruguay* or uzbek* or vanuatu* or new hebrides* or venezuela* or vietnam* or viet nam* or yemen* or yugoslav* or zambia* or zimbabwe* or rhodesia* or arab* countr* or middle east* or global south or sahara* or subsahara* or magreb* or maghrib* or west indies* or caribbean* or central america* or latin america* or south america* or central asia* or north asia* or northern asia* or southeastern asia* or south eastern asia* or southeast asia* or south east asia* or west asia* or western asia* or east europe* or eastern europe* or developing countr* or developing nation* or developing population* or developing world or less developed countr* or less developed nation* or less developed world or lesser developed countr* or lesser developed nation* or lesser developed world or under developed countr* or under developed nation* or under developed world or underdeveloped countr* or underdeveloped nation* or underdeveloped world or middle income countr* or middle income nation* or middle income population* or low income countr* or low income nation* or low income population* or lower income countr* or lower income nation* or lower income population* or underserved countr* or underserved nation* or underserved population* or under served population* or under served nation* or under served population* or deprived countr* or deprived population* or high burden countr* or high burden nation* or countdown countr* or countdown nation* or poor countr* or poor nation* or poor population* or poor world or poorer countr* or poorer nation* or poorer population* or poorer world or developing econom* or less developed econom* or underdeveloped econom* or under developed econom* or middle income econom* or low income econom* or lower income econom* or low gdp or low gnp or low gross domestic or low gross national or lower gdp or lower gnp or lower gross domestic or lower gross national or lmic or lmics or third world or lami countr* or transitional countr* or emerging econom* or emerging nation*).ti,ab,kf.
16. 5 and 9 and 14 and 15
