# Supplementary material for: Understanding the Gendered Impact of COVID-19 on Young Self-Employed Nigerian Women and Coproducing Interventions That Foster Better Systems and Well-Being: Protocol for a Multimethods Study
Source: JMIR Res Protoc. 2025 May 30;14:e69577. doi: 10.2196/69577 (PMC12166318; doi:10.2196/69577)
Supplement: Multimedia Appendix 4 [file resprot_v14i1e69577_app4.docx]

**Systematic Review Search Strategy from Medline via Ovid**

1. Entrepreneurship/
2. ("Self-employed worker*" or "Freelance*" or "Entrepreneur*" or "Business owner*" or "CEO*" or "Chief Executive Officer*" or "self-employed").ti,ab,kf.
3. ("Self-employed wom*" or "Entrepreneur* wom*" or "Female Chief Executive Officer*" or "Female business owner" or "Female entrepreneur*" or "Business women" or "Female CEO*").ti,ab,kf.
4. ("self-employed men" or "Entrepreneur* men" or "Male entrepreneur*" or "Male Chief Executive Officer*" or "male business owner" or "Business men" or "Male CEO*").ti,ab,kf.
5. 1 or 2 or 3 or 4
6. Crisis Intervention/ or Internet-Based Intervention/ or Psychosocial Intervention/
7. Policy/ or Public Policy/
8. ("Intervention*" or "program*" or "initiative*" or "Active Labour Market Program*" or ("intervention*" adj3 "co-design*") or ("program*" adj3 "co-design*") or ("initiative*" adj3 "co-design*") or ("Active Labour Market Program*" adj3 "co-design*") or ("Active Labor Market Program*" adj3 "co-design*") or "Active Labor Market Program*" or ("intervention*" adj3 "co-produc*")).ti,ab,kf.
9. (("program*" adj3 "co-produc*") or ("initiative*" adj3 "co-produc*") or ("Active Labour Market Program*" adj3 "co-produc*") or ("Active Labor Market Program*" adj3 "co-produc*") or "policy" or "policies" or ("Policy" adj3 "co-design*") or ("Policies" adj3 "co-design*")).ti,ab,kf.
10. 6 or 7 or 8 or 9
11. ("job quality" or "quality of work" or "income" or "earning*" or "profit*" or "work* hour*" or "Work-life balance" or "prospect*" or "Skill use and discretion" or "Level of skill*" or "Physical environment" or "Work hazard*" or "Work intensity" or "Job fulfilment" or "Job satisfaction" or "Decent work" or "Quality of Employment" or "Work Quality").ti,ab,kf.
12. Mental Health/ or "Quality of Life"/
13. ("Mental wellbeing" or "Mental well-being" or "Social wellbeing" or "Social well-being" or "Physical wellbeing" or "Physical well-being" or "Mental health" or "Physical health" or "Social connectedness" or "Social health" or "Happiness" or "Job satisfaction" or "Well-being" or "well being" or "wellbeing" or "Wellness" or "life satisfaction" or "Resilien*" or "Self-efficacy" or "Tenaci*" or "quality of life" or "positive affect").ti,ab,kf.
14. ("coping" or "Coping strateg*" or "coping skill*" or "Coping style*" or "Coping mechanism*" or "Coping behav*" or "cope").ti,ab,kf.
15. 11 or 12 or 13 or 14
16. 5 and 10 and 15
17. limit 16 to yr="2003 -Current"
