# Supplementary material for: Understanding the Gendered Impact of COVID-19 on Young Self-Employed Nigerian Women and Coproducing Interventions That Foster Better Systems and Well-Being: Protocol for a Multimethods Study
Source: JMIR Res Protoc. 2025 May 30;14:e69577. doi: 10.2196/69577 (PMC12166318; doi:10.2196/69577)
Supplement: Multimedia Appendix 6 [file resprot_v14i1e69577_app6.pdf]

IDRC · CRDI
International Development Research Centre
Centre de recherches pour le développement international

July 29, 2022

Subject: Congratulations - Your Women RISE application is selected for grant negotiation.

Dear Olayinka Omigbodun, Srividya Iyer, and Olafunmilayo Akinpelu,

On behalf of Women RISE donors, it is my pleasure to inform you that your project entitled 'Understanding the Gendered Impact of COVID-19 on Young Self-Employed Nigerian Women and Co-Producing Solutions that Foster Better Systems and Wellbeing' (IDRC Ref #110029) has been recommended by the external scientific review committee to proceed to the grant negotiation stage. Congratulations to you, and your team for writing a strong proposal that scored well to reach this stage of IDRC granting process. We look forward to our collaboration. In the coming weeks we shall be finalizing the grant agreements to be sent to your LMIC based institution and Canada based institution. Please note that the two grant agreements need to be synchronous and one cannot take effect without the other one. As per the call for proposals, the anticipated start date of the grant is October 1st, 2022, and the grant duration cannot exceed 24 months, ending on September 30, 2024.

In order to be able to complete this granting process in a timely manner, we will require your prompt and due diligence over the next weeks to finalize the documentation and budget. I would like to take this opportunity to introduce you to Anne-Marie Butuba (email address below) who will be in touch regarding additional documentation requirements.

Please submit this material as soon as possible and no later than 5 August 16:59 EDT. Kindly note that if the requested documents are not received by this date your prospective grant may instead be given to the next high ranking research team, we apologise for this strict procedure. Please note that this message is not considered a final approval of the funds for the project and no expenses can be incurred until all grant agreements are executed by the collaborating institutions.

Your application benefitted from external reviewers' feedback which we share below as an annex to this message.

The responsible Program Officer will be in touch with you in due course to discuss how this feedback can be taken into account during the course of the planning and implementation of your project, after the successful conclusion of the grant negotiations.
If you have any questions related to this granting process, please do not hesitate to contact the responsible
Program Officer, Montasser Kamal (mkamal@idrc.ca), or Grants Officer, Anne-Marie Butuba abutuba@idrc.ca, and we will be happy to respond.
Gmail - Congratulations - Your Women RISE application is selected for grant negotiation 19/02/2025, 12:23

Once again congratulations and we look forward to working with you on this exciting project.
Sincerely,
John

John Dusabe-Richards, PhD
Director, Global Health | Directeur, Santé mondiale
IDRC | CRDI
150 Kent St, Ottawa, ON K1P 0B2, Canada
Tel: +1-613-696-2203 | jdusabe-richards@idrc.ca
www.idrc.ca | www.crdi.ca
Subscribe IDRC Bulletin | Abonnement Bulletin du CRDI

Annex
Application No WR-0000000327
Summary of External Reviewer Committee Feedback

1. Rationale and originality
   -This proposal is well written and organised. The need for research to understand how self-employed women in Nigeria fared during COVID is strongly justified with academic and grey literature. (There is no reference for the study by Kusi- Mensah, however).
   -The research has some unique elements compared to other projects which tend to focus on women exclusively and see men and families as barriers to be overcome; this project in contrast positions families (including fathers and husbands) and networks as potential points of support to be strengthened.

2. Research design and feasibility
   - The questions are clearly articulated and clearly laid out. The components seem well thought out and likely to generate interesting and useful findings. It takes a nuanced and realistic approach to gender relations. The extensive secondary data review is super important.
   - In terms of weaknesses: the digital story telling sounds interesting but is not well described. The interviews will also yield data, but the proposal does not say how this data or the digital story telling data fit with the focus groups mentioned later at the Intervention stage; how will that

data be used to inform the intervention, especially as it seems that the agenda for the intervention has already been set by workshop already held?

- More concerns -- not insurmountable -- not enough detail on how the Interventions will be implemented, by whom, and no mention of possible challenges, problems.
- Consider how you will include a diverse group of women as research participants.

3. Gender equality and inclusion considerations

-The proposal addresses the priorities of the Women RISE call. The proposal reflects a more nuanced understanding of gender relations than most and this is reflected in the design of the research which included comparison with men who are self employed and includes an understand that women have networks and families who include men.
-The proposal includes an understanding that participation in the research and intervention can build capacity for women. Women participants are consulted at every stage.

4. Research Team capacity

- The RT seems extremely well rounded, interdisciplinary, and strong with the expertise, experience and networks to conduct the research and develop and evaluate the intervention.

5. Knowledge mobilization and impact

-There is no doubt that the interviews, digital story telling, academic and secondary literature review will generate insightful and useful knowledge.
- It is not clear that the intervention can be implemented and evaluated in such a short time, nor what the outcomes will be. It is not guaranteed that they will be successful and be taken up and scaled up. What kind of impact can realistically be seen within this timeframe?
